# Functional properties of flagellin as a stimulator of innate immunity

**DOI:** 10.1038/srep18379

**Published:** 2016-01-12

**Authors:** Yuan Lu, James R. Swartz

**Affiliations:** 1 Department of Chemical Engineering, Stanford University, Stanford, CA 94305; 2 Department of Bioengineering, Stanford University, Stanford, CA 94305.

## Abstract

We report the development of a well-defined flagellin-based nanoparticle stimulator and also provide a new mechanism of action model explaining how flagellin-triggered innate immunity has evolved to favor localized rather than potentially debilitating systemic immune stimulation. Cell-free protein synthesis (CFPS) was used to facilitate mutational analysis and precisely orientated display of flagellin on Hepatitis B core (HBc) protein virus-like particles (VLPs). The need for product stability and an understanding of mechanism of action motivated investigations indicating that the D0 domain of flagellin is sensitive to amino acid sequence independent hydrolysis – apparently due to the need for structural flexibility during natural flagellin polymerization. When D0-stabilized flagellin was attached to HBc VLPs with the D0 domain facing outward, flagellin’s tendency to polymerize caused the VLPs to precipitate. However, attaching the D0 domain to the VLP surface produced a stable nanoparticle adjuvant. Surprisingly, attaching only 2 flagellins per VLP provided the same 1 pM potency as did VLPs with about 33 attached flagellins suggesting that the TLR5 receptor is highly effective in delivering its intracellular signal. These observations suggest that flagellin’s protease sensitivity, tendency to aggregate, and very high affinity for TLR5 receptors limit its systemic distribution to favor localized immune stimulation.

Flagellin, a principal component of bacterial flagella, stimulates host defense in a variety of organisms, including mammals, insects, and plants^1^. As a natural agonist of human toll-like receptor 5 (TLR5), flagellin activates the innate immune response, which is considered important for priming and regulating the adaptive immune response^2^. Over the past several years, a strong interest has emerged in developing flagellin as an adjuvant for use in human vaccines to stimulate humoral and cell-mediated immune responses^3^. For example, VaxInnate is now testing antigen-flagellin fusion proteins in clinical trials^4^. However, flagellin is both difficult to produce with high quality and is unstable^5^. These observations suggested that flagellin’s mechanisms of action needed further investigation. Moreover, as for any injectable, it is highly desirable that flagellin is well-defined, consistently manufactured, and stable during preparation, storage, and administration.

Recently an *Escherichia coli*-based *in vitro* cell-free protein synthesis (CFPS) method was developed to rapidly produce soluble flagellin protein^5^. Currently, flagellin is mainly produced by *in vivo* recombinant DNA technology, and most evaluations of flagellin as an immune stimulator have used one of the two forms of flagellin from *Salmonella typhimurium*, FliC and FljB^6^,^7^,^8^. CFPS technology is emerging as a powerful platform for the synthesis of pharmaceutical proteins^9^,^10^,^11^,^12^ and can produce proteins from either PCR products or plasmid templates in a few hours. The open nature of the CFPS system allows facile modification of the reaction environment, and the absence of a cell wall enables simpler purification procedures^13^. Especially when flagellin is urgently needed for new and highly potent vaccines at short notice, for example to combat pandemic influenza threats, CFPS can provide rapid, cost-effective and high-yielding production^13^.

However, the C-terminal domain of flagellin is susceptible to proteolytic degradation. The flagellin protein consists of four globular domains (D0, D1, D2 and D3), with the TLR5 recognition site in the conserved D1 domain^6^. In previous work, many different flagellin EC50’s (concentrations which produce 50% of maximal bioactivity) have been reported ranging from 20 pM to 2 nM^14^,^15^,^16^,^17^,^18^. This wide variation may be caused by the C-terminal portion of the D0 domain being attacked by proteases^5^. Although the D0 domain is relatively far from the TLR5 recognition region, D0 domain deletion significantly reduced flagellin’s bioactivity. The CFPS platform allowed us to add protease inhibitors to inhibit the proteolysis and obtain intact flagellin. We therefore hypothesized that the D0 domain of monomeric flagellin is unstructured rendering it susceptible to random proteases and peptidases. We further hypothesized that introducing an intrinsic structural feature, i.e., a disulfide bond, could stabilize the D0 domain and help prevent proteolysis.

We also proposed that a flagellin-displaying nanoparticle would enhance the stability and bioactivity of flagellin. A preferable approach is to array multiple flagellin molecules on the surface of virus-like particles (VLPs) with an orientation optimized for TLR5 recognition. VLPs offer precisely defined nanometer-scale scaffolds^19^ that are non-infectious. With repetitive surfaces that can display a high density of molecules, they have been extensively explored as nanoparticle vehicles for many applications in biotechnology (e.g., vaccines, drug delivery). Among different types of VLPs, the Hepatitis B core (HBc) protein VLP is currently the most promising model for fundamental and applied immunological studies^20^. The size of the HBc VLP makes it ideal for trafficking to lymph nodes where robust protective responses can be elicited^21^.

The CFPS platform provides a facile means for introducing non-natural amino acids (nnAAs) with an alkyne moiety into flagellin and with an azide moiety on the outer surface of the VLP. This allows for direct flagellin-VLP coupling using Cu(I)-catalyzed azide-alkyne cycloaddition, the “click” reaction^22^,^23^. In our previous work, flagellin presentation on the VLP surface increased specific TLR5 stimulation activity by approximately 10-fold^5^. However, the display of flagellin on VLPs by attachment through the tip of the D3 domain decreased the solubility of the resultant nanoparticles indicating that the conjugation sites in flagellin need to be optimized. In addition, further characterization of this modular nanoparticle stimulator was needed, in particular, the effect of flagellin surface density.

Here we report the use of alanine scanning, multiple mutations, and different *E. coli* cell extracts to analyze and avoid the proteolysis of flagellin in the CFPS system. The cell extract made from *E. coli* BL21 inflicted less proteolysis, but the CFPS yield was low. Increasing the concentration of the chaperone GroEL/S improved the yield greatly. New disulfide bonds were then introduced in or near the D0 domain to stabilize this protease sensitive region of flagellin. To improve the functional properties of VLP-flagellin conjugates, different nnAA sites near the N-terminus of flagellin or at the distal end of the D3 domain were tested. Finally, to investigate why flagellins displayed on VLPs induced higher bioactivity, different numbers of flagellins were displayed on VLPs producing somewhat surprising results.

## Results

### Analysis of flagellin proteolysis

**Figure 1:**
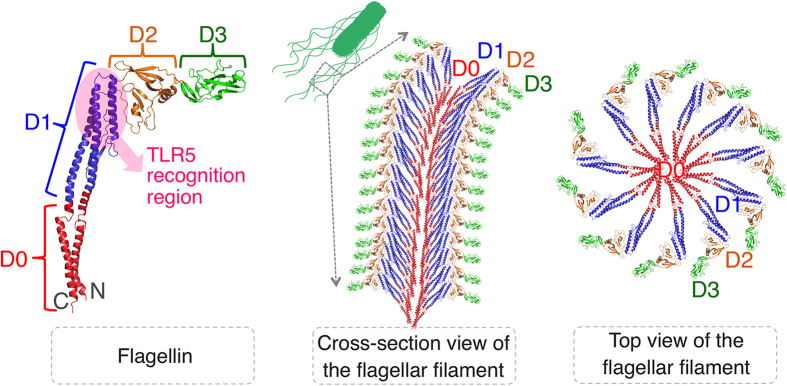
The structure of flagellin and the cross-sectional and top views of the flagellar filament. The flagellar filament is composed of a single protein, flagellin. Note that the TLR5 recognition region is not accessible in flagella.

The flagellin (FliC) protein as an immune stimulator was successfully synthesized in a CFPS system using *E. coli* KC6 extract^5^. The flagellin accumulated as a soluble protein to ~300 μg/mL. However, SDS-PAGE autoradiogram analysis with and without C-terminal Strep II tag purification showed that flagellin accumulated partially as a C-terminally truncated form. The full-length flagellin protein has a molecular mass of 52.7 kDa, and the main truncated product is approximately 47 kDa. C-terminal degradation of flagellin occurs not only in the CFPS system but also with *in vivo* production^24^. The addition of protease inhibitors in the CFPS system confirmed that the truncation was due to proteolysis even though KC6 extract has been used for the synthesis of many different proteins with only rare proteolysis. For flagellin’s bacterial function, flagellin self-polymerizes to form tubular bacterial flagella (Fig. 1). We hypothesized that the D0 domain of flagellin was more easily attacked by proteases because it is loosely structured to provide conformational malleability during polymerization (Fig. 1). By further analysis using protein purification and mass spectrometry, the C-terminal helix of the D0 domain was confirmed as the protease target, and cleavage at the R453 position was suggested as a frequent occurrence (see Supplementary Fig. S1 online)^5^.

**Figure 2:**
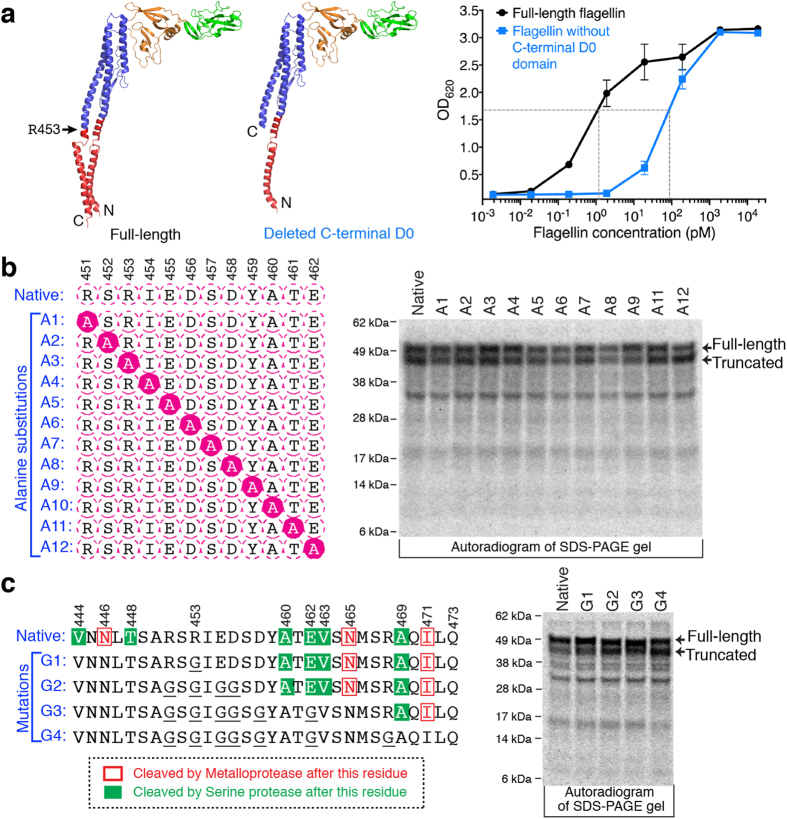
Analysis of flagellin proteolysis. (**a**) Bioactivity assay of full-length flagellin and flagellin without the C-terminal D0 domain (S452-R495). Five measurements were taken on different days. (**b**) SDS-PAGE autoradiogram analysis of alanine-scanning mutants after CFPS reactions. The native sequence is the same as version A10. (**c**) Prediction of cleavage sites in the flagellin sequence (444-473) using PROSPER server, design of four mutations (mutated residues underlined) and SDS-PAGE autoradiogram analysis of CFPS products. The CFPS was conducted at 30 °C for 6 h, using *E. coli* KC6 extract.

An alternative option was to produce flagellin without the C-terminal D0 domain (S452-R495). However, deletion of this domain increased the EC50 value to 80 pM from 1.3 pM (Fig. 2a), indicating a 60-fold reduction in bioactivity. Consequently, we focused on understanding and preventing the proteolysis.

Alanine scanning near position R453 was employed in an attempt to identify specific peptide bonds subject to flagellin proteolysis. To determine the importance of individual residues, we introduced 11 mutations (A1 through A9, A11, and A12; A10 is unmutated) near R453 (Fig. 2b). The GCG codon was used. SDS-PAGE analysis showed that none of the CFPS products had diminished truncation, suggesting that the individual amino acid residues around R453 are not critical for proteolysis.

In an alternative approach, possible protease cleavage sites near R453 were predicted using PROSPER web-server^25^, which is capable of predicting cleavage sites for 24 different protease families within a single substrate sequence. The flagellin sequence between residues 444 and 473 was evaluated (Fig. 2c). The potential for serine protease family cleavage was suggested after residues (P1 position) V444, T448, A460, E462, V463, and A469. In addition, metalloprotease cleavage was predicted after residues N446, N465 and I471. Based on this analysis and the previously observed benefit of protease inhibitors, we mutated these possible cleavage sites to avoid proteolysis. Because charged amino acid residues in proteins often are the cleavage targets of proteases^26^,^27^, the charged amino acids (R, E, D) in the sequence 444–473 were mutated to glycine (codon GGC). Four mutants (G1, G2, G3 and G4) were designed (Fig. 2c). PROSPER evaluation suggested that these 4 sets of mutations would greatly reduce the proteolytic cleavage. However, SDS-PAGE analysis showed that these CFPS-produced mutants still accumulated with a large fraction of truncated product (Fig. 2c). Based on the failure of both alanine scanning and targeted mutagenesis to reduce the proteolysis and based on the natural flagellin folding and polymerization process, we concluded that the truncations are related to a lack of consistent structure in the D0 domain of monomeric flagellin.

### Avoiding flagellin proteolysis

**Figure 3:**
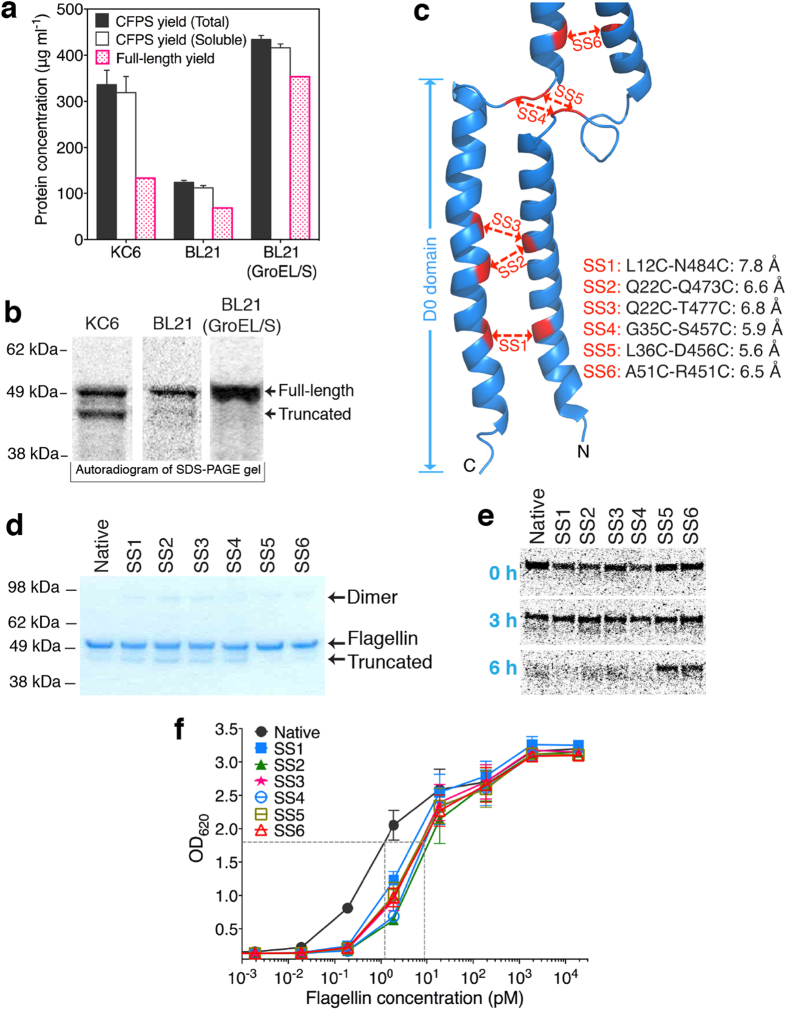
Inhibition of flagellin proteolysis by domain stabilization. (**a**) CFPS total and full-length yields and (**b**) SDS-PAGE autoradiogram analysis of flagellin produced by 6 h reactions using three different *E. coli* extracts (KC6 extract, BL21 extract, and BL21 extract with overexpressed GroEL/S). (**c**) Positions of newly introduced disulfide bonds (SS1 though SS6) at or near the D0 domain. (**d**) Non-reducing SDS-PAGE analysis of flagellin after purification and oxidation by 20 mM diamide for 3 hours. The flagellin mutants were synthesized using CFPS with BL21 extract containing overexpressed GroEL/S. (**e**) Stability analysis (by SDS PAGE) of flagellin mutants SS1 through SS6) produced by CFPS using the KC6 extract. The purified and oxidized flagellin mutants were incubated in a CFPS mixture at 30 °C for 6 h and analyzed by autoradiography at 0, 3, and 6 h. (**f**) Bioactivity of flagellin mutants. Five measurements were taken on different days; error bars indicate the standard error of the mean (SEM).

Since the intrinsically disordered conformation of flagellin near the C-terminus appeared to be the main reason for protease cleavage, we next evaluated cell extracts deficient in the major proteases. Previous studies indicated that the Lon protease might be partially responsible^5^. Lon proteases are ATP-dependent serine proteinases^28^ belonging to the MEROPS peptidase family S16. The putative function of the Lon protease in bacteria is thought to be the degradation of unfolded proteins and therefore Lon might be expected to attack a disordered D0 domain^29^. *E. coli* BL21 is deficient in Lon protease activity, and CFPS of flagellin using an *E. coli* BL21 cell extract avoided most of the truncated product. Unfortunately, however, the full-length yield was reduced to only 70 μg/mL (Fig. 3a,b).

Suspecting impaired protein folding, we next evaluated the effect of increasing the concentration of the GroEL-GroES (GroEL/S) chaperonin^30^,^31^. *E. coli* BL21 cell extract with overexpressed GroEL/S^9^ provided a 5-fold yield increase (from 70 μg/mL to 350 μg/mL, Fig. 3a) while avoiding truncated flagellin accumulation (Fig. 3b). Newly synthesized polypeptide chains of cytosolic proteins have the potential to begin folding co-translationally^32^,^33^. The chaperonin GroEL/S not only functions to avoid aggregation for some proteins, but also can accelerate folding substantially^31^ which could also improve the translation of the flagellin mRNA while discouraging proteolysis.

Although, we had achieved good flagellin production levels with minimal truncation, we were still concerned about product stability during preparation, storage, and administration. We reasoned that stability could be improved by introducing disulfide bonds at or near the D0 domain to mimic the natural stabilization conferred by flagellin polymerization. We replaced native amino acids with cysteine at the following locations (Fig. 3c): L12-N484 (SS1); Q22-Q473 (SS2); Q22-T477 (SS3); G35-S457 (SS4); L36-D456 (SS5); and A51-R451 (SS6). Native flagellin and mutants were synthesized in the CFPS system using BL21 extract with overexpressed GroEL/S and were then purified using Strep-tactin resin. Oxidation by 20 mM diamide at room temperature for 3 h was used to encourage new disulfide bond formation. SDS-PAGE analysis (Fig. 3d) showed that small amounts of dimers formed after diamide treatment for some mutants (as might be expected for a domain with weak structural integrity). After purification and oxidation, small amounts of native, SS1, SS2, SS3, and SS4 were truncated, but the SS5 and SS6 mutants had minimal dimer formation as well as minimal truncation (Fig. 3d). To analyze the stability of flagellin mutants SS1 to SS6, the purified and oxidized flagellin proteins (20 μg/ml) were added to the CFPS system with KC6 extract. After 6 h of incubation at 30 °C, all the flagellin proteins suffered truncations but mutants SS5 and SS6 were significantly stabilized (Fig. 3e). However, cell-based TLR5 stimulation assays showed that introducing these disulfide bonds decreased bioactivities (Fig. 3f). The disulfide bridge apparently changes the conformation of the receptor recognition surface in the D1 domain. Even so, these flagellin mutants were still highly bioactive (EC50 < 10 pM) relative to EC50 values reported for recombinant flagellin ranging from 20 pM to 2 nM^14^,^15^,^16^,^17^,^18^. Based on the above analysis, mutant SS5 was used in subsequent development.

### Conjugation of flagellin to VLPs

To enhance the TLR5 receptor activation, flagellin proteins were presented in an ordered array on the surface of the disulfide bond-stabilized HBc VLP using site-specific click conjugation (see Supplementary Fig. S2 online)^22^,^34^.

**Figure 4:**
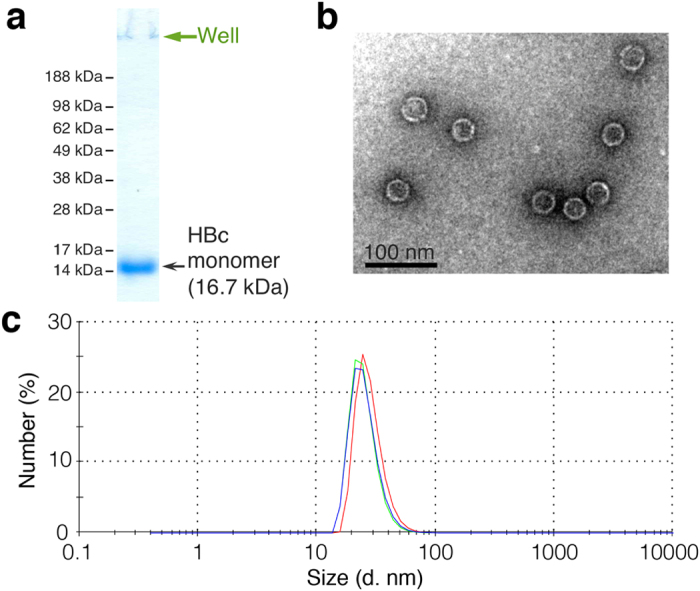
Characteristics of HBc VLPs. (**a**) Purity analysis by reducing SDS-PAGE. (**b**) VLP size determination using transmission electron microscopy (TEM). (**c**) VLP analysis by dynamic light scattering (DLS).

HBc proteins were first synthesized in the CFPS system, purified by ammonium sulfate precipitation, and then self-assembled by adding 10 mM Tris-HCl buffer (pH 7.4) with 0.5 M NaCl. After size-exclusion chromatography (SEC), HBc VLPs with high purity (Fig. 4a) were obtained. The VLP size was then determined by transmission electron microscopy (TEM) and dynamic light scattering (DLS). The average diameter of HBc VLPs was estimated as 30 nm by TEM analysis (Fig. 4b) and as 26 nm by DLS analysis (Fig. 4c). The disulfide bond-stabilized HBc VLPs showed very good stability during preparation and storage.

**Figure 5:**
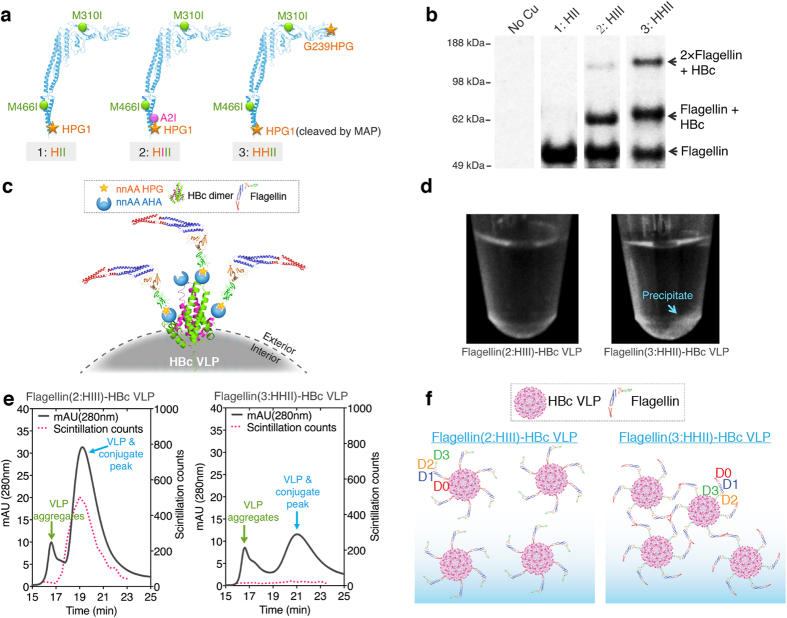
Selection of nnAA attachment sites in flagellin. (**a**) Three-dimensional structures of flagellin showing the mutation positions of three flagellin mutants (HII, HPG1+M310I+M466I; HIII, HPG1+A2I+M310I+M466I; and HHII, HPG1+G239HPG+M310I+M466I). (**b**) Reducing SDS-PAGE autoradiogram analysis of the click reaction products after attaching flagellin mutants to HBc VLPs. The molar ratio of flagellin (52.7 kDa) to HBc monomer (16.7 kDa) in the reactions was 1:2. (**c**) Diagram of HHII flagellins conjugated to HBc VLPs. There are two nnAA AHAs in each HBc monomer: at the N-terminus and at the 76 site. (**d**) Photographs showing precipitation of the click reaction products after HHII attachment to the VLP. (**e**) Size-exclusion HPLC profiles of the click-reaction supernatants with radioactive flagellin and non-radioactive HBc VLPs. (**f**) Diagrams suggesting a probable cause for the HHII flagellin-HBc conjugate precipitation.

NnAAs can be successfully incorporated into CFPS-produced flagellin using global methionine replacement^23^. In this scheme, a CFPS reaction is conducted in which methionine is replaced by the methionine analogue homopropargylglycine (HPG) to present an alkyne moiety. Two HPG sites, M1 at the N-terminus and G239 at the distal end of the D3 domain were individually evaluated. Both were expected to display flagellin on the VLP surface with exposure of the TLR5 binding region (Fig. 5a). The two native methionines in flagellin, M310 (D2 domain) and M466 (D0 domain), were mutated to isoleucine to prevent HPG incorporation at these sites (Fig. 5a).

To ensure attachment at the distal end of the D3 domain, we needed to remove the M1 HPG required to initiate translation, and we took advantage of a naturally occurring enzyme called Methionine AminoPeptidase (MAP). In *E. coli*, MAP performs the essential post-translational N-terminal methionine excision of nascent polypeptides during protein synthesis^35^,^36^. The excision is heavily dependent on the amino acid in the second position and is most efficient if that amino acid is Ala, Gly or Ser^37^. The amino acid in the second position of native flagellin sequence is Ala. Click-reaction results (Fig. 5b) showed that flagellin mutant HII (HPG1+M310I+M466I) could not be attached onto VLPs, suggesting that the N-terminal methionine analogue, HPG, was efficiently excised by the MAP in the cell extract. We therefore created two flagellin mutants (Fig. 5a). One was HIII (HPG1+A2I+M310I+M466I) to produce a flagellin with only the N-terminal HPG. The second amino acid Ala was mutated to Ile to discourage HPG removal by MAP^37^. The second mutant was HHII (HPG1+G239HPG+M310I+M466I) with only one HPG at the distal end of the D3 domain. The N-terminal HPG was assumed to be removed by MAP as previously inferred.

The flagellin mutant HIII with the N-terminal HPG and mutant HHII with HPG only at the distal end of the D3 domain were first compared. The mutant HHII showed better conjugation efficiency with HBc VLPs (Fig. 5b). Because there were two AHAs in each HBc monomer, we observed both one and two HHII flagellins attached to HBc monomers (Fig. 5c). However, the flagellin(HHII)-HBc VLP conjugates were prone to precipitation (Fig. 5d), which was further confirmed by size-exclusion HPLC analysis of the click-reaction supernatants (Fig. 5e). When the HHII mutant flagellin is attached to the VLPs, the D0 domains are exposed. It appears that intersubunit hydrophobic interactions of the D0 domains cause the flagellin(HHII)-HBc VLP conjugates to aggregate and precipitate (Fig. 5f). Therefore, the flagellin mutant (HIII) with only the N-terminal HPG was used in subsequent development.

**Figure 6:**
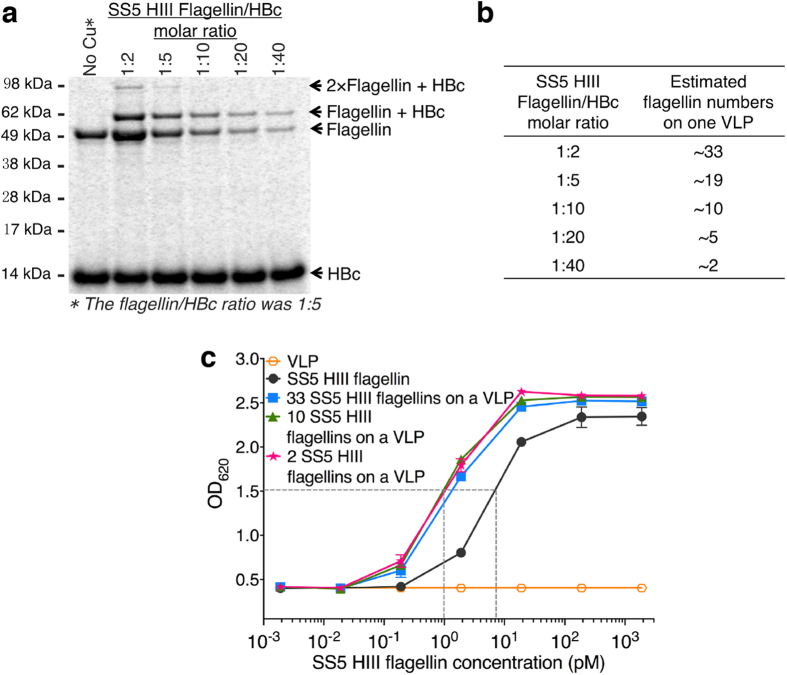
Attachment of SS5 HIII flagellin to VLPs. (**a**) Reducing SDS-PAGE autoradiogram analysis after click reactions of flagellin with HBc VLPs. The molar ratios of flagellin (52.7 kDa) to HBc monomer (16.7 kDa) in the reaction mixture were respectively 1:2, 1:5, 1:10, 1:20, and 1:40. (**b**) Estimated flagellin numbers attached to each VLP were determined by gel densitometry. (**c**) Bioactivity analysis of flagellin-HBc VLP conjugates produced using the 1:2, 1:10, and 1:40 reactant ratios. The data were measured in triplicate; error bars indicate the standard error of the mean (SEM).

In previous work, we had shown that flagellin has about 10 times higher bioactivity when presented in an ordered array on a VLP^5^. The final step in developing VLP conjugated flagellin as an immunostimulant was therefore to evaluate the effects of varying the number of flagellin molecules per VLP. Although we expected to see an avidity effect, the very high affinity to TLR5 made this study of interest. The click conjugation was therefore performed using different molar ratios of flagellin to HBc monomer (1:2, 1:5, 1:10, 1:20, and 1:40). The concentration of HBc monomer was kept constant at 20 μM. The highest surface loading obtained was 33 flagellin proteins per HBc VLP (120 dimer spikes) using the 1:2 ratio as determined by the densitometric analysis (Fig. 6a,b). The bioactivities of the flagellin-VLP conjugates with various numbers of flagellins (33, 10, or 2 flagellins on a VLP) suggested that the VLP-conjugated flagellin was approximately 7-fold more active than free flagellin in stimulating the TLR5 receptor (Fig. 6c). Surprisingly, there was no significant bioactivity difference between VLPs with various numbers of flagellins. We hypothesize that relatively few TLR5 binding events may be sufficient for robust cell stimulation. The saturation analysis of TLR5 binding with soluble SS5 HIII flagellin (Fig. 6c) showed that the minimum flagellin concentration stimulating the TLR5 receptors was very low at 0.2 pM to support this hypothesis. Although some VLPs had many more flagellin molecules, fewer of those VLPs were added to the cell culture to achieve the same flagellin concentration. In all cases, considering rotational as well as spatial diffusion, the frequency that a VLP-bound flagellin will encounter a TLR5 receptor would be about the same. Most likely a single VLP could only bind to at most two cells because of steric exclusion. Thus most of the multiple flagellin copies on a VLP would not have a chance to conjugate to a TLR5. However, an avidity effect may still have allowed equivalent responses from fewer VLPs.

Nonetheless the improved bioactivity for the attached flagellin is consistent with free flagellin becoming partially inactivated by forming soluble aggregates in the aqueous solution through the interactions of D0 domains. The TLR5 recognition sites would be blocked or less effective in such aggregates. The oriented N-terminal attachment of flagellin (mutant HIII) to the VLPs ensured that the D0 domain was not available for aggregation.

## Discussion

Recognizing the need for effective and well-characterized vaccine adjuvants, we focused on the innate immunity TLR5 agonist, flagellin. Because of its unusual post-translational localization and folding pathway, this protein has been difficult to produce by conventional technologies as evidenced by the wide diversity in reported potencies^14^,^15^,^16^,^17^,^18^. In contrast, the dilute and accessible nature of cell-free protein synthesis reactions enabled the consistent production of the most potent reported flagellin. This technology also allowed the rapid diagnosis of the cause of product truncation during production. The use of alternative and engineered cell extracts avoided the proteolysis and dramatically improved product yields.

Because product stability is also a concern during purification, storage, and administration; we used mutagenesis to assess the cause of the proteolysis. We discovered that the D0 domain is hydrolyzed in an apparently sequence independent manner. We suggest that this results from a lack of conformational stability in this domain; most likely required for the natural localization and maturation mechanisms that allow this unusual protein to form a long flagellum. As shown in Fig. 1, the two helices of the D0 domain of flagellin interact with each other and with other D0 domains to form the core structure of flagella. This occurs after flagellin has been secreted outward down the core channel of flagella to self-polymerize at the growing tip of the flagellum. During this process, the possibly disordered D0 domain of flagellin would be protected from proteases. The polymerization would then confer a protease resistant defined structure. Introducing disulfide bonds between the two protein strands that join the D1 and D0 domains reduced the proteolysis of unpolymerized flagellin, presumably by stabilizing this region of the protein. This stabilized version of flagellin provides an important new research reagent for gaining better insights into innate immunity stimulation.

To develop potentially more effective immune stimulators, we next attached the stabilized flagellin to HBc VLPs using specific attachment sites on the flagellin to evaluate the influences of orientation and flagellin surface concentration on nanoparticle solution stability and TLR5 stimulation potency. Display of the stabilized D0 domain resulted in nanoparticle precipitation that was avoided when the D0 domain was attached to the VLP surface. Surprisingly, attaching only about 2 flagellins per VLP provided the same 1 pM (based on flagellin concentration) potency as did VLPs with about 33 attached flagellins. These results suggest that the TLR5 receptor is highly effective in delivering its intracellular signal.

**Figure 7:**
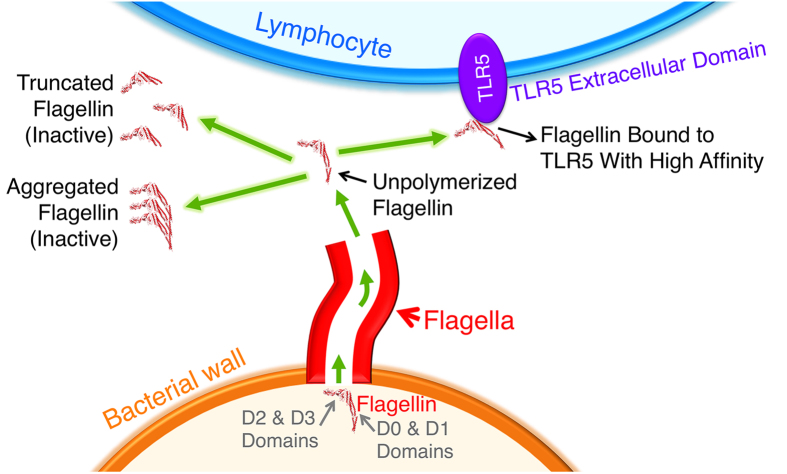
Proposed model that suggests flagellin innate immune response stimulation is limited to the immediate location of early bacterial infections.

The observations enabled by these new reagents suggest that flagellin’s protease sensitivity, tendency to aggregate, and very high affinity for the TLR5 receptor will limit the systemic distribution of the flagellin released by the invading bacteria, at least for early, low cell density infections. Flagellin that fails to polymerize onto the growing tip of the flagellum will be released from the bacterium as a monomer (Fig. 7). However, the D0 domains will still tend to polymerize (aggregate) and, if not, will be disordered and subject to proteolysis. Both aggregation and C-terminal hydrolysis reduce flagellin potency by 10- to 100-fold. In addition, the very high affinity of intact flagellin to TLR5 receptors on local lymphocytes will minimize its escape from the local area. The increase in local TLR5 concentration resulting from lymphocyte recruitment will further limit flagellin release into circulation. We suggest that this is an evolved feature of such innate immune responses in order to allow effective inactivation of frequent bacterial invasions without inducing the potentially debilitating effects of major systemic responses.

In summary, these studies produced new reagents not previously available, a modified and stabilized form of flagellin and a VLP nanoparticle that presents flagellin in an effective orientation. An immune stimulator consisting of stabilized flagellin attached to the VLP through its D0 domain will be protected from proteolysis as well as aggregation. With this well-characterized nanoparticle stimulator, reliable *in vivo* studies can now be pursued to assess pharmacokinetic performance and, more importantly, stimulation efficacy as a function of the number of flagellin molecules per VLP. In addition, it may also be beneficial to evaluate modifications of the TLR5 affinity by introducing targeted D1 domain mutations. We expect that such an optimized immune stimulator will enable optimal delivery to lymph nodes while still enhancing strong, targeted stimulation of B and T cell responses.

## Methods

### Construction of vectors for CFPS reactions

The flagellin gene (*flic*) from *S. typhimurium* SL1344 (GenBank Accession No. CBW17983) was cloned into the pY71 vector using NdeI and SalI restriction sites as described previously^5^. pY71 is a plasmid (1.76 kb) that utilizes the T7 promoter and contains a pUC19 origin of replication and a kanamycin resistance element^38^. The coding sequence for a C-terminal *Strep*-tag II purification tag was also added. The gene sequence encoding the human Hepatitis B core (HBc) antigen of subtype adyw (UniProt accession number: P03147) with the C-terminus truncated at amino acid 149 was optimized for *E. coli* tRNA concentrations. The vector pET24a-HBc149 was generated by ligation of the optimized HBc protein gene into the pET-24a(+) vector (Novagen, San Diego, CA) at the NdeI and XhoI restriction sites. The sequences of flagellin and HBc constructs can be found as Supplementary Table S1 and Table S2 online.

### Cell-Free Protein Synthesis (CFPS)

CFPS was conducted using the PANOx-SP (PEP, amino acids, nicotinamide adenine dinucleotide (NAD), oxalic acid, spermidine, and putrescine) cell-free system as described previously^39^ with several modifications. The standard PANOx-SP CFPS reaction mixture includes: 1.2 mM ATP, 0.85 mM each of GTP, UTP, and CTP, 33 mM phosphoenol pyruvate (Roche Molecular Biochemicals, Indianapolis, IN), 170 mM potassium glutamate, 10 mM ammonium glutamate, 16 mM magnesium glutamate, 1.5 mM spermidine, 1.0 mM putrescine, 0.17 mg/mL folinic acid, 13.3 μg/mL plasmid, approximately 100−300 μg/mL T7 RNA polymerase, 2 mM of each of the 20 unlabeled amino acids, 0.33 mM NAD, 0.26 mM Coenzyme A (CoA), 2.7 mM potassium oxalate, and 0.28 volumes of *E. coli* KC6 S30 extract^40^. To increase the CFPS yield using BL21 extract, the GroEL-GroES (GroEL/S) chaperonin was overexpressed in BL21 cells before preparing the extract^9^.

CFPS reactions to produce the flagellin and HBc proteins were conducted at 30 °C for 6 h. Small-scale CFPS reactions were carried out in 20 μL volumes in 1.5 mL microcentrifuge tubes. Preparative-scale reactions used 3 mL volumes with 1 mL per well in 6-well tissue culture plates (BD, Franklin Lakes, NJ). 8.4 μM L-[U−^14^C]-Leucine (PerkinElmer, Waltham, MA) was added to small-scale reactions and to 20 μL aliquots of preparative-scale reactions for measuring protein yields using a previously described trichloroacetic acid protein precipitation protocol^41^ and a Beckman LS3801 liquid scintillation counter (Beckman Coulter, Fullerton, CA). The soluble fraction of preparative-scale reactions was recovered by centrifugation at 21,000 × g for 15 min for further evaluation and purification.

Protein size was analyzed by SDS-PAGE gel and autoradiography. NuPAGE Novex precast gels and reagents were purchased from Invitrogen (Carlsbad, CA). For reducing SDS-PAGE, samples were denatured for 5 min at 95 °C in loading buffer (1X LDS running buffer and 50 mM dithiothreitol). For non-reducing SDS-PAGE, samples were only mixed with LDS running buffer without addition of dithiothreitol and heat treatment. The samples were loaded onto a 10% (w/v) Bis-Tris precast gel using the SeeBlue Plus2 molecular weight standard, and the electrophoresis was conducted using the MES running buffer (Invitrogen). SimplyBlue SafeStain (Invitrogen) was used to stain and fix the gels according to the manufacturer’s recommendations. The gels were dried using a gel dryer model 583 (Bio-Rad, Richmond, CA), before exposure to a storage phosphor screen (Molecular Dynamics), which was subsequently scanned using a Typhoon Scanner (GE Healthcare). ImageJ software^42^ was used to compare the density of bands on the gel to determine the yield of full-length protein.

### Purification of Flagellin Proteins

Soluble CFPS products from 3 mL reactions were purified using *Strep*-tag II/*Strep*-tactin affinity chromatography (IBA Gmbh, Gottingen, Germany). The pH of soluble fractions was adjusted to 8.0 and the solutions were applied to a 1.0 mL Strep-Tactin gravity flow column (IBA Gmbh) and washed with 10 mL of 100 mM Tris-HCl buffer (pH 8.0) containing 150 mM NaCl. The loaded column was eluted with PBS buffer containing 5.0 mM desthiobiotin, and 0.5 mL fractions were analyzed for protein content using SDS-PAGE gels. Pooled fractions were then dialyzed against PBS buffer to remove the desthiobiotin and stored at 4 °C for further analysis.

### Purification of HBc VLPs

The CFPS products were centrifuged for 15 min at 15,000 g to remove aggregates. After collecting the supernatant, saturated ammonium sulfate was added dropwise while mixing to a final concentration of 1.2 M. The supernatant was mixed for an additional 10 min at room temperature and centrifuged at 10,000 × g to pellet the precipitate. The precipitate was resuspended in Dialysis Buffer (10 mM Tris-HCl, pH 7.4, 0.5 M NaCl) with 10 mM dithiothreitol (DTT) by vortexing. The mixture was then dialyzed against Dialysis Buffer with 1 mM DTT to remove residual ammonium sulfate and centrifuged for 15 min at 15,000 g to remove aggregates. The supernatant was loaded onto a Sepharose 6 Fast Flow column pre-equilibrated with the same buffer, and fractions were collected for analysis by SDS-PAGE. The VLP fractions were pooled and concentrated by ultrafiltration using an Amicon stirred cell with a 3 kDa molecular weight cutoff regenerated cellulose membrane. VLP fractions were then oxidized to form disulfide bonds by adding 20 mM diamide. The oxidants were removed by dialysis against the Dialysis Buffer with five rounds of buffer exchange. The endotoxins in VLP solutions were then removed by phase separation using 0.5% Triton X-114.

### Transmission electron microscopy (TEM)

A 5 μL sample of a purified 5 nM VLP solution was applied to a carbon coated copper/Formvar grid and negatively stained with 1% w/v uranyl acetate, pH 4. Photographs were taken with a Gatan Orius CCD camera in a JEOL JEM1400 electron microscope at 120 kV acceleration voltage.

### Dynamic light scattering (DLS)

DLS measurements were performed with a Malvern Zetasizer Nano ZS instrument (Malvern Instruments Ltd, Worcestershire, UK). The software used to collect and analyze the data was Zetasizer Series version 6.12 from Malvern. Fifty μl of each sample was analyzed in a disposable solvent resistant micro cuvette (ZEN0040) (Malvern Instruments Ltd, Worcestershire, UK). The measurements were made at a controlled temperature of 25 °C. For each sample, 15 runs of 10 s were performed with three repetitions. The size distributions were obtained from the Zetasizer software.

### Constructs used to produce flagellin for conjugation to virus-like particles (VLPs)

For the site-specific incorporation of non-natural amino acids (nnAAs) with an alkyne moiety in flagellin, the method of global methionine replacement was evaluated. M310 and M466 were replaced with isoleucine residues to avoid nnAA introduction at these two sites using Quikchange PCR. One mM of the methionine analogue homopropargylglycine (HPG) (Chiralix B.V., Nijmegen, The Netherlands) with an alkyne moiety was added to CFPS reactions. For these reactions, methionine was omitted.

To display an azide moiety on the surface of Hepatitis B core antigen (HBc) VLPs, a methionine codon (ATG) was introduced using QuikChange PCR to encode residue 76 for nnAA incorporation. The M66 codon was replaced with a serine codon (AGC) to avoid nnAA introduction at this site. One mM of the methionine analogue azidohomoalanine (AHA) (MedChem Source LLP, Federal Way, WA) with an azide moiety was added to CFPS reactions. Methionine was also omitted from these CFPS reaction mixtures. HBc VLPs were stabilized by introducing a SS1(D29C-R127C) disulfide bond network^43^.

### Azide-Alkyne cycloaddition and purification

The azide-alkyne cycloaddition reactions were conducted in an anaerobic glovebox (Coy Laboratories, Grass Lake, MI) to preserve the reduced state of the tetrakis(acetonitrile)copper(I)hexafluorophosphate catalyst ([(CH_3_CN)_4_Cu]PF_6_ or simply Cu(I) catalyst) (Sigma Aldrich, St. Louis, MO). Cu(I) catalyst was added to reactions at 2 mM. HBc VLP and flagellin were mixed with the Cu (I) catalyst in 20 mM potassium phosphate (pH 8.0) with 0.01% Tween 20. Before addition of the Cu(I) catalyst, click reaction components were deoxygenated in 1.5 mL microcentrifuge tubes for 1 h in the anaerobic glovebox. The click reactions for attaching flagellin to the HBc VLP were conducted for 2 h.

Conjugated HBc VLP-flagellin assemblies were then analyzed using SDS-PAGE and purified by size exclusion chromatography using an Ultrahydrogel 500 HPLC column (30 cm × 7.8 mm inner diameter with 10 μM particles) (Waters). The running buffer was PBS (pH 7.4) with 0.01% Tween 20, pumped at 0.3 mL/min. The injection volume was 90 μL. Protein absorbance was monitored in-line at 280 nm over a period of 60 min.

### Flagellin bioactivity assay

Flagellin bioactivity was analyzed using HEK-Blue™-hTLR5 cells (Invivogen) as described in Lu *et al.*, (2013), which had been generated by co-transfection of the human TLR5 gene and an inducible SEAP reporter gene into HEK293 cells. Firstly, a cell suspension of fresh HEK-Blue™-hTLR5 Cells was prepared at ~140,000 cells per ml in medium containing 10% (v/v) heat-inactivated FBS. Then, 20 μL of the flagellin sample was mixed with 180 μl of cell suspension (~25,000 cells) per well in a sterile flat-bottom 96-well plate, and the plate was incubated at 37 °C in a CO_2_ incubator for 24 h. After that, 20 μL of induced HEK-Blue™-hTLR5 cell supernatant was mixed with 180 μL of resuspended QUANTI-Blue™ per well of a flat-bottom 96-well plate and incubated at 37 °C for 2 h. The relative SEAP concentrations were determined by absorbance at 620 nm using a VersaMax microplate reader.

## Additional Information

**How to cite this article**: Lu, Y. and Swartz, J. R. Functional properties of flagellin as a stimulator of innate immunity. *Sci. Rep.* **6**, 18379; doi: 10.1038/srep18379 (2016).

## Supplementary Material

Supplementary Information
