# Supplementary material for: Functional properties of flagellin as a stimulator of innate immunity
Source: Sci Rep. 2016 Jan 12;6:18379. doi: 10.1038/srep18379 (PMC4709591; doi:10.1038/srep18379)
Supplement: Supplementary Information [file srep18379-s1.pdf]

SUPPLEMENTARY INFORMATION

Functional properties of flagellin as a stimulator of innate immunity

Yuan Lu[1] and James R. Swartz[1, 2, *]

1. Department of Chemical Engineering, Stanford University, Stanford, CA 94305

2. Department of Bioengineering, Stanford University, Stanford, CA 94305

[*] Correspondence to James R. Swartz, Department of Chemical Engineering, Stanford University, 381 North-South Mall, Stanford, CA 94305-5025, USA; email: jswartz@stanford.edu; telephone: 650-723-5398; fax: 650-725-0555

Supplementary Tables

Supplementary Table S1. The sequences of different flagellin variants.

Variants	Protein sequence	DNA encoding sequence
Wild-type	MAQVINTNSLSLLTQNNLNKSQSAL<br>GTAIERLSSGLRINSAKDDAAGQAI<br>ANRFTANIKGLTQASRNANDGISIA<br>QTTEGALNEINNNLQRVRELAVQSA<br>NSTNSQSDLDSIQAEITQRLNEIDR<br>VSGQTQFNGVKVLAQDNTLTIQVGA<br>NDGETIDIDLKQINSQTLGLDTLNV<br>QQKYKVSDTAATVTGYADTTIALDN<br>STFKASATGLGGTDQKIDGDLKFDD<br>TTGKYYAKVTVTGGTGKDGYYEVSV<br>DKTNGEVTLAGGATSPLTGGLPATA<br>TEDVKNVQVANADLTEAKAALTAAG<br>VTGTASVVKMSYTDNNGKTIDGGLA<br>VKVGDDYYSATQNKDGSISINTTKY<br>TADDGTSKTALNKLGGADGKTEVVS<br>IGGKTYAASKAEGHNFKAQPDLAEA<br>AATTTENPLQKIDAALAQVDTLRSD<br>LGAVQNRFNSAITNLGNTVNNLTSA<br>RSRIEDSDYATEVSNMSRAQILQQA<br>GTSVLAQANQVPQNVLSLLR**WSHPQ**<br>**FEK**	ATGGCACAAGTGATTAATACAAACAGCCTGTCGCTGTTGACCCAGAATAACCTGAACAAATCCCAGTCCGCTC<br>TGGGCACCGCTATCGAGCGTCTGTCTTCCGGTCTGCGTATCAACAGCGCGAAAGACGATGCGGCAGGTCAGGC<br>GATTGCTAACCGTTTTACCGCGAACATCAAAGGTCTGACTCAGGCTTCCCGTAACGCTAACGACGGTATCTCC<br>ATTGCGCAGACCACTGAAGGCGCGCTGAACGAAATCAACAACAACCTGCAGCGTGTGCGTGAACTGGCGGTT<br>CAGTCTGCTAACAGCACCAACTCCCAGTCTGACCTCGACTCCATCCAGGCTGAAATCACCCAGCGCCTGAACG<br>AAATCGACCGTGTATCCGGCCAGACTCAGTTCAACGGCGTGAAAGTCCTGGCGCAGGACAACACCCTGACCA<br>TCCAGGTTGGTGCCAACGACGGTGAAACTATCGATATCGATCTGAAGCAGATCAACTCTCAGACCCTGGGTCT<br>GGATACGCTGAATGTGCAACAAAAATATAAGGTCAGCGATACGGCTGCAACTGTTACAGGATATGCCGATAC<br>TACGATTGCTTTAGACAATAGTACTTTTAAAGCCTCGGCTACTGGTCTTGGTGGTACTGACCAGAAAATTGATG<br>GCGATTTAAAATTTGATGATACGACTGGAAAATATTACGCCAAAGTTACCGTTACGGGGGGAACTGGTAAAG<br>ATGGCTATTATGAAGTTTCCGTTGATAAGACGAACGGTGAGGTGACTCTTGCTGGCGGTGCGACTTCCCCGCT<br>TACAGGTGGACTACCTGCGACAGCAACTGAGGATGTGAAAAATGTACAAGTTGCAAATGCTGATTTGACAGA<br>GGCTAAAGCCGCATTGACAGCAGCAGGTGTTACCGGCACAGCATCTGTTGTTAAGATGTCTTATACTGATAAT<br>AACGGTAAAACTATTGATGGTGGTTTAGCAGTTAAGGTAGGCGATGATTACTATTCTGCAACTCAAAATAAAG<br>ATGGTTCCATAAGTATTAATACTACGAAATACACTGCAGATGACGGTACATCCAAAACTGCACTAAACAAACT<br>GGGTGGCGCAGACGGCAAAACCGAAGTTGTTTCTATTGGTGGTAAAACTTACGCTGCAAGTAAAGCCGAAGG<br>TCACAACTTTAAAGCACAGCCTGATCTGGCGGAAGCGGCTGCTACAACCACCGAAAACCCGCTGCAGAAAAT<br>TGATGCTGCTTTGGCACAGGTTGACACGTTACGTTCTGACCTGGGTGCGGTACAGAACCGTTTCAACTCCGCTA<br>TTACCAACCTGGGCAACACCGTAAACAACCTGACTTCTGCCCGTAGCCGTATCGAAGATTCCGACTACGCGAC<br>CGAAGTTTCCAACATGTCTCGCGCGCAGATTCTGCAGCAGGCCGGTACCTCCGTTCTGGCGCAGGCGAACCAG<br>GTTCCGCAAAACGTCCTCTCTTTACTGCGT**TGGAGCCATCCGCAGTTTGAAAAA**TAA
HII	**(HPG)**AQVINTNSLSLLTQNNLNKS<br>QSALGTAIERLSSGLRINSAKDDAA<br>GQAIANRFTANIKGLTQASRNANDG<br>ISIAQTTEGALNEINNNLQRVRELA<br>VQSANSTNSQSDLDSIQAEITQRLN<br>EIDRVSGQTQFNGVKVLAQDNTLTI<br>QVGANDGETIDIDLKQINSQTLGLD<br>TLNVQQKYKVSDTAATVTGYADTTI<br>ALDNSTFKASATGLGGTDQKIDGDL<br>KFDDTTGKYYAKVTVTGGTGKDGYY<br>EVSVDKTNGEVTLAGGATSPLTGGL<br>PATATEDVKNVQVANADLTEAKAAL<br>TAAGVTGTASVVK**I**SYTDNNGKTID<br>GGLAVKVGDDYYSATQNKDGSISIN<br>TTKYTADDGTSKTALNKLGGADGKT<br>EVVSIGGKTYAASKAEGHNFKAQPD<br>LAEAAATTTENPLQKIDAALAQVDT<br>LRSDLGAVQNRFNSAITNLGNTVNN<br>LTSARSRIEDSDYATEVSN**I**SRAQI<br>LQQAGTSVLAQANQVPQNVLSLLR**W**<br>**SHPQFEK**	**ATG**GCACAAGTGATTAATACAAACAGCCTGTCGCTGTTGACCCAGAATAACCTGAACAAATCCCAGTCCGCTC<br>TGGGCACCGCTATCGAGCGTCTGTCTTCCGGTCTGCGTATCAACAGCGCGAAAGACGATGCGGCAGGTCAGGC<br>GATTGCTAACCGTTTTACCGCGAACATCAAAGGTCTGACTCAGGCTTCCCGTAACGCTAACGACGGTATCTCC<br>ATTGCGCAGACCACTGAAGGCGCGCTGAACGAAATCAACAACAACCTGCAGCGTGTGCGTGAACTGGCGGTT<br>CAGTCTGCTAACAGCACCAACTCCCAGTCTGACCTCGACTCCATCCAGGCTGAAATCACCCAGCGCCTGAACG<br>AAATCGACCGTGTATCCGGCCAGACTCAGTTCAACGGCGTGAAAGTCCTGGCGCAGGACAACACCCTGACCA<br>TCCAGGTTGGTGCCAACGACGGTGAAACTATCGATATCGATCTGAAGCAGATCAACTCTCAGACCCTGGGTCT<br>GGATACGCTGAATGTGCAACAAAAATATAAGGTCAGCGATACGGCTGCAACTGTTACAGGATATGCCGATAC<br>TACGATTGCTTTAGACAATAGTACTTTTAAAGCCTCGGCTACTGGTCTTGGTGGTACTGACCAGAAAATTGATG<br>GCGATTTAAAATTTGATGATACGACTGGAAAATATTACGCCAAAGTTACCGTTACGGGGGGAACTGGTAAAG<br>ATGGCTATTATGAAGTTTCCGTTGATAAGACGAACGGTGAGGTGACTCTTGCTGGCGGTGCGACTTCCCCGCT<br>TACAGGTGGACTACCTGCGACAGCAACTGAGGATGTGAAAAATGTACAAGTTGCAAATGCTGATTTGACAGA<br>GGCTAAAGCCGCATTGACAGCAGCAGGTGTTACCGGCACAGCATCTGTTGTTAAG**ATC**TCTTATACTGATAAT<br>AACGGTAAAACTATTGATGGTGGTTTAGCAGTTAAGGTAGGCGATGATTACTATTCTGCAACTCAAAATAAAG<br>ATGGTTCCATAAGTATTAATACTACGAAATACACTGCAGATGACGGTACATCCAAAACTGCACTAAACAAACT<br>GGGTGGCGCAGACGGCAAAACCGAAGTTGTTTCTATTGGTGGTAAAACTTACGCTGCAAGTAAAGCCGAAGG<br>TCACAACTTTAAAGCACAGCCTGATCTGGCGGAAGCGGCTGCTACAACCACCGAAAACCCGCTGCAGAAAAT<br>TGATGCTGCTTTGGCACAGGTTGACACGTTACGTTCTGACCTGGGTGCGGTACAGAACCGTTTCAACTCCGCTA<br>TTACCAACCTGGGCAACACCGTAAACAACCTGACTTCTGCCCGTAGCCGTATCGAAGATTCCGACTACGCGAC<br>CGAAGTTTCCAAC**ATC**TCTCGCGCGCAGATTCTGCAGCAGGCCGGTACCTCCGTTCTGGCGCAGGCGAACCAG<br>GTTCCGCAAAACGTCCTCTCTTTACTGCGT**TGGAGCCATCCGCAGTTTGAAAAA**TAA
HIII	**(HPG)**IQVINTNSLSLLTQNNLNKS<br>QSALGTAIERLSSGLRINSAKDDAA<br>GQAIANRFTANIKGLTQASRNANDG<br>ISIAQTTEGALNEINNNLQRVRELA<br>VQSANSTNSQSDLDSIQAEITQRLN<br>EIDRVSGQTQFNGVKVLAQDNTLTI<br>QVGANDGETIDIDLKQINSQTLGLD<br>TLNVQQKYKVSDTAATVTGYADTTI<br>ALDNSTFKASATGLGGTDQKIDGDL<br>KFDDTTGKYYAKVTVTGGTGKDGYY<br>EVSVDKTNGEVTLAGGATSPLTGGL<br>PATATEDVKNVQVANADLTEAKAAL<br>TAAGVTGTASVVK**I**SYTDNNGKTID<br>GGLAVKVGDDYYSATQNKDGSISIN<br>TTKYTADDGTSKTALNKLGGADGKT<br>EVVSIGGKTYAASKAEGHNFKAQPD<br>LAEAAATTTENPLQKIDAALAQVDT<br>LRSDLGAVQNRFNSAITNLGNTVNN<br>LTSARSRIEDSDYATEVSN**I**SRAQI<br>LQQAGTSVLAQANQVPQNVLSLLR**W**<br>**SHPQFEK**	**ATG**ATACAAGTGATTAATACAAACAGCCTGTCGCTGTTGACCCAGAATAACCTGAACAAATCCCAGTCCGCTC<br>TGGGCACCGCTATCGAGCGTCTGTCTTCCGGTCTGCGTATCAACAGCGCGAAAGACGATGCGGCAGGTCAGGC<br>GATTGCTAACCGTTTTACCGCGAACATCAAAGGTCTGACTCAGGCTTCCCGTAACGCTAACGACGGTATCTCC<br>ATTGCGCAGACCACTGAAGGCGCGCTGAACGAAATCAACAACAACCTGCAGCGTGTGCGTGAACTGGCGGTT<br>CAGTCTGCTAACAGCACCAACTCCCAGTCTGACCTCGACTCCATCCAGGCTGAAATCACCCAGCGCCTGAACG<br>AAATCGACCGTGTATCCGGCCAGACTCAGTTCAACGGCGTGAAAGTCCTGGCGCAGGACAACACCCTGACCA<br>TCCAGGTTGGTGCCAACGACGGTGAAACTATCGATATCGATCTGAAGCAGATCAACTCTCAGACCCTGGGTCT<br>GGATACGCTGAATGTGCAACAAAAATATAAGGTCAGCGATACGGCTGCAACTGTTACAGGATATGCCGATAC<br>TACGATTGCTTTAGACAATAGTACTTTTAAAGCCTCGGCTACTGGTCTTGGTGGTACTGACCAGAAAATTGATG<br>GCGATTTAAAATTTGATGATACGACTGGAAAATATTACGCCAAAGTTACCGTTACGGGGGGAACTGGTAAAG<br>ATGGCTATTATGAAGTTTCCGTTGATAAGACGAACGGTGAGGTGACTCTTGCTGGCGGTGCGACTTCCCCGCT<br>TACAGGTGGACTACCTGCGACAGCAACTGAGGATGTGAAAAATGTACAAGTTGCAAATGCTGATTTGACAGA<br>GGCTAAAGCCGCATTGACAGCAGCAGGTGTTACCGGCACAGCATCTGTTGTTAAG**ATC**TCTTATACTGATAAT<br>AACGGTAAAACTATTGATGGTGGTTTAGCAGTTAAGGTAGGCGATGATTACTATTCTGCAACTCAAAATAAAG<br>ATGGTTCCATAAGTATTAATACTACGAAATACACTGCAGATGACGGTACATCCAAAACTGCACTAAACAAACT<br>GGGTGGCGCAGACGGCAAAACCGAAGTTGTTTCTATTGGTGGTAAAACTTACGCTGCAAGTAAAGCCGAAGG<br>TCACAACTTTAAAGCACAGCCTGATCTGGCGGAAGCGGCTGCTACAACCACCGAAAACCCGCTGCAGAAAAT<br>TGATGCTGCTTTGGCACAGGTTGACACGTTACGTTCTGACCTGGGTGCGGTACAGAACCGTTTCAACTCCGCTA<br>TTACCAACCTGGGCAACACCGTAAACAACCTGACTTCTGCCCGTAGCCGTATCGAAGATTCCGACTACGCGAC<br>CGAAGTTTCCAAC**ATC**TCTCGCGCGCAGATTCTGCAGCAGGCCGGTACCTCCGTTCTGGCGCAGGCGAACCAG<br>GTTCCGCAAAACGTCCTCTCTTTACTGCGT**TGGAGCCATCCGCAGTTTGAAAAA**TAA

**HHII**	**(HPG)**AQVINTNSLSLLTQNNLNKS<br>QSALGTAIERLSSGLRINSAKDDAA<br>GQAIANRFTANIKGLTQASRNANDG<br>ISIAQTTEGALNEINNNLQRVRELA<br>VQSANSTNSQSDLDSIQAEITQRLN<br>EIDRVSGQTQFNGVKVLAQDNTLTI<br>QVGANDGETIDIDLKQINSQTLGLD<br>TLNVQQKYKVSDTAATVTGYADTTI<br>ALDNSTFKASATGLGGTDQKIDGDL<br>KFDDTTGKYYAKVTVTG**(HPG)**TGK<br>DGYYEVSVDKTNGEVTLAGGATSPL<br>TGGLPATATEDVKNVQVANADLTEA<br>KAALTAAGVTGTASVVK**I**SYTDNNG<br>KTIDGGLAVKVGDDYYSATQNKDGS<br>ISINTTKYTADDGTSKTALNKLGGA<br>DGKTEVVSIGGKTYAASKAEGHNFK<br>AQPDLAEAAATTTENPLQKIDAALA<br>QVDTLRSDLGAVQNRFNSAITNLGN<br>TVNNLTSARSRIEDSDYATEVSN**I**S<br>RAQILQQAGTSVLAQANQVPQNVLS<br>LLR**WSHPQFEK**	**ATG**GCACAAGTGATTAATACAAACAGCCTGTCGCTGTTGACCCAGAATAACCTGAACAAATCCCAGTCCGCTC<br>TGGGCACCGCTATCGAGCGTCTGTCTTCCGGTCTGCGTATCAACAGCGCGAAAGACGATGCGGCAGGTCAGGC<br>GATTGCTAACCGTTTTACCGCGAACATCAAAGGTCTGACTCAGGCTTCCCGTAACGCTAACGACGGTATCTCC<br>ATTGCGCAGACCACTGAAGGCGCGCTGAACGAAATCAACAACAACCTGCAGCGTGTGCGTGAACTGGCGGTT<br>CAGTCTGCTAACAGCACCAACTCCCAGTCTGACCTCGACTCCATCCAGGCTGAAATCACCCAGCGCCTGAACG<br>AAATCGACCGTGTATCCGGCCAGACTCAGTTCAACGGCGTGAAAGTCCTGGCGCAGGACAACACCCTGACCA<br>TCCAGGTTGGTGCCAACGACGGTGAAACTATCGATATCGATCTGAAGCAGATCAACTCTCAGACCCTGGGTCT<br>GGATACGCTGAATGTGCAACAAAAATATAAGGTCAGCGATACGGCTGCAACTGTTACAGGATATGCCGATAC<br>TACGATTGCTTTAGACAATAGTACTTTTAAAGCCTCGGCTACTGGTCTTGGTGGTACTGACCAGAAAATTGATG<br>GCGATTTAAAATTTGATGATACGACTGGAAAATATTACGCCAAAGTTACCGTTACGGGG**ATG**ACTGGTAAAG<br>ATGGCTATTATGAAGTTTCCGTTGATAAGACGAACGGTGAGGTGACTCTTGCTGGCGGTGCGACTTCCCCGCT<br>TACAGGTGGACTACCTGCGACAGCAACTGAGGATGTGAAAAATGTACAAGTTGCAAATGCTGATTTGACAGA<br>GGCTAAAGCCGCATTGACAGCAGCAGGTGTTACCGGCACAGCATCTGTTGTTAAG**ATC**TCTTATACTGATAAT<br>AACGGTAAAACTATTGATGGTGGTTTAGCAGTTAAGGTAGGCGATGATTACTATTCTGCAACTCAAAATAAAG<br>ATGGTTCCATAAGTATTAATACTACGAAATACACTGCAGATGACGGTACATCCAAAACTGCACTAAACAAACT<br>GGGTGGCGCAGACGGCAAAACCGAAGTTGTTTCTATTGGTGGTAAAACTTACGCTGCAAGTAAAGCCGAAGG<br>TCACAACTTTAAAGCACAGCCTGATCTGGCGGAAGCGGCTGCTACAACCACCGAAAACCCGCTGCAGAAAAT<br>TGATGCTGCTTTGGCACAGGTTGACACGTTACGTTCTGACCTGGGTGCGGTACAGAACCGTTTCAACTCCGCTA<br>TTACCAACCTGGGCAACACCGTAAACAACCTGACTTCTGCCCGTAGCCGTATCGAAGATTCCGACTACGCGAC<br>CGAAGTTTCCAAC**ATC**TCTCGCGCGCAGATTCTGCAGCAGGCCGGTACCTCCGTTCTGGCGCAGGCGAACCAG<br>GTTCCGCAAAACGTCCTCTCTTTACTGCGT**TGGAGCCATCCGCAGTTTGAAAAA**TAA

CAPS represent the flagellin. **Underlined BOLD CAPS** represent the *Strep*-tag II polypeptide. The mutations were marked in red color.

Supplementary Table S2. The sequence of HBc protein.

Variant	Protein sequence	DNA encoding sequence
**HBc**	MDIDPYKEFGATVELLSFLPSDFFP<br>SVR**C**LLDTAAALYRDALESPEHCSP<br>HHTALRQAILCWGDL**S**TLATWVGTN<br>**(AHA)**EDPASRDLVVSYVNTNVGLK<br>FRQLLWFHISCLTFGRETVLEYLVS<br>FGVWI**C**TPPAYRPPNAPILSTLPET<br>TVV	ATGGATATCGACCCGTACAAAGAATTCGGCGCGACCGTTGAACTGCTGTCTTTCCTGCCGTCT<br>GATTTCTTCCCGTCTGTTCGT**TGC**CTGCTGGACACCGCGGCAGCACTGTACCGTGACGCGCTG<br>GAATCTCCGGAACATTGTTCTCCGCATCACACTGCGCTGCGTCAGGCGATTCTGTGCTGGGGC<br>GACCTGagcACCCTGGCGACTTGGGTTGGCACCAACatgGAAGATCCGGCGTCTCGTGATCTGG<br>TTGTTTCTTACGTTAACACTAACGTTGGTCTGAAATTCCGTCAGCTGCTGTGGTTCCACATCTC<br>TTGCCTGACCTTCGGTCGTGAAACCGTTCTGGAATACCTGGTTTCTTTTGGTGTTTGGATT**TGT**<br>ACTCCGCCGGCTTACCGTCCGCCGAACGCACCGATCCTGAGCACCCTGCCGGAAACCACTGTT<br>GTGTAATAA

CAPS represent the HBc protein. **Underlined** represent the mutations. The mutations for introducing a disulfide bond network were marked in red color.

Supplementary Figures

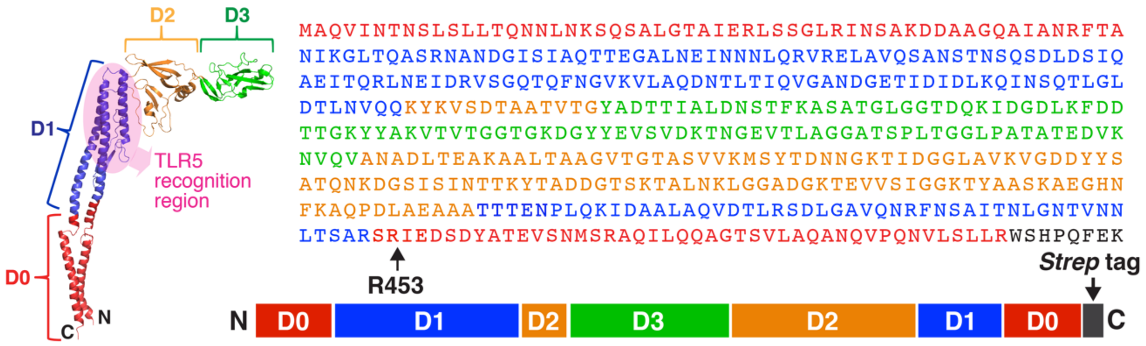

Supplementary Figure S1. Positions of flagellin domains (D0, D1, D2 and D3) in amino acid sequence and possible truncation position (R453).

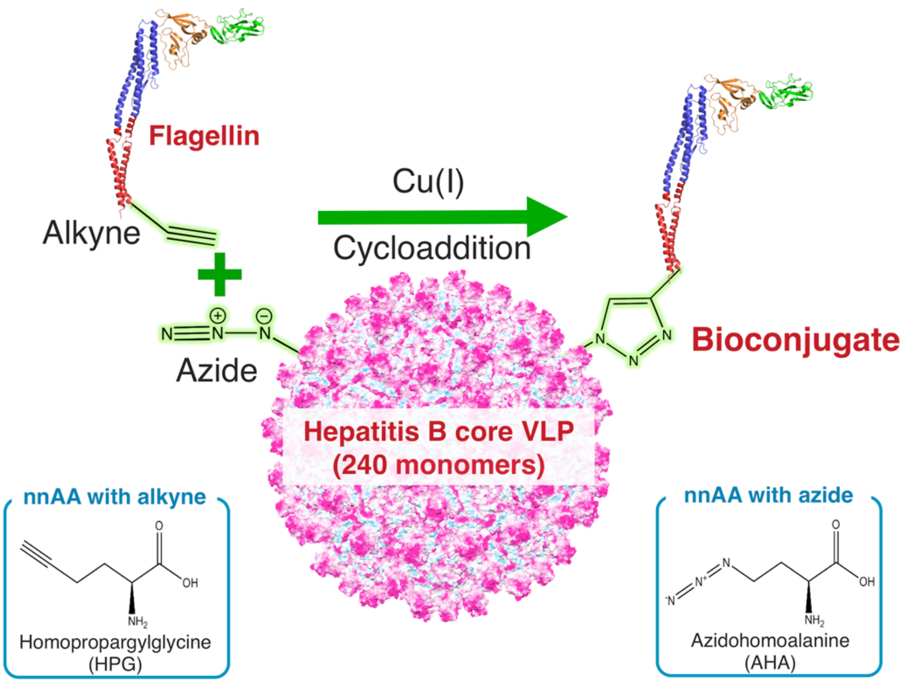

Supplementary Figure S2. Diagram of Cu(I)-catalyzed [3 + 2] cycloaddition click chemistry reaction for the direct coupling of flagellin proteins to HBc VLPs. The HBc VLP is stabilized by introducing a SS1(D29C-R127C) disulfide bond network[1].

Reference

1. Lu, Y., Chan, W., Ko, B. Y., VanLang, C. C. & Swartz, J. R. Assessing sequence plasticity of a virus-like nanoparticle by evolution toward a versatile scaffold for vaccines and drug delivery. *Proc. Natl. Acad. Sci. USA* **112**, 12360-12365 (2015).
